# Fabrication of polyester fabrics with tungsten bronze nanorods and a silane coupling agent for improved thermal storage and washing durability

**DOI:** 10.1186/s40691-022-00310-y

**Published:** 2023-01-05

**Authors:** Ye-eun Woo, Kyung Wha Oh

**Affiliations:** 1 Dept of Fashion, Graduate School, Chung-Ang University, Seoul, 06974 Republic of Korea; 2 Dept. of Fashion, College of Art, Chung-Ang University, Anseong, 17546 Republic of Korea

**Keywords:** Tungsten bronze nanorods, Outdoor sportswear, Silane coupling agent, Photothermal effect, Washing durability

## Abstract

The thermal storage and washing durability of polyester fabrics treated with tungsten bronze nanorods(TBNRs) were analyzed to determine the optimal concentration for the photothermal effect in this study. TBNRs with an average length of 34.0 ± 2.5 nm and a diameter of 2.3 ± 0.4 nm were synthesized by the thermal decomposition of Ammonium metatungstate hydrate(AMT) in oleylamine (OA) to generate TBNRs that are capable of emitting heat by efficiently absorbing light in the near-infrared region. The effect of TBNR concentration and the silane coupling agent on the photothermal effect and washing durability of the PET fabric were evaluated with a solar simulator. As a result, as the concentration of TBNRs increased, the photothermal effect increased, and the maximum photothermal effect was shown at 5 wt%. In addition, washing durability were further improved by adding 0.5 wt% silane counpling agent. Overall, the post-processing treatment effectively increased the photothermal effect without a significant change in the physical properties and color of the polyester.

## Introduction

Owing to the recent spread of Covid-19, consumers have come to spend more time in the natural environment while maintaining a safe distance from each other. As outdoor activities increased, demand for outdoor sportswear has also increased (Han, 2021). In addition, the rapid changes in climate and the environment have fundamentally changed the way people dress (Bae, 2011), which has led to more interests in the thermal insulation of textile products. Thus, fibers and fabrics with properties such as light weight, thermal insulation, and heat storage have been developed (Koo et al., 2007). The most recently used thermal insulation method have done by adding various ceramics (Choe et al., 2006), but there is a limit to the thermal insulation capacity of these fabrics in extreme environments. Therefore, this study aimed to improve the thermal insulation of polyester fabrics by treating the fabric with nanoparticles that have a light heating property (photothermal effect) in an outdoor environment.

Photothermal materials, including conductive, semiconductor, and magnetic materials, such as tungsten bronze and graphene oxide, absorb energy and convert it into heat when irradiated with long-wavelength near-infrared (NIR, 780–3000 nm) waves. These materials have a lower energy level than ultraviolet or visible light; hence, they can absorb NIR waves, with long wavelengths, which are harmless to the human body (Jeon et al., 2019). Tungsten trioxide (WO_3_) is not suitable for NIR absorption; however, since alkali metal ions (M = Li + , Na + , K + , Cs +) are incorporated into the crystal structure of WO_3_, a part of the W^6+^ in the crystal is reduced to W^5+^ to form a conduction band. Sub-bands are created in the plasmon region, forming localized surface plasmon resonance (LSPR) and sub-band transitions. These enable the reduced WO_3_ and MxWO_3_ materials to strongly absorb NIR and release heat. (Park, 2020). As a result, tungsten bronze nanoparticles such as tungsten trioxide doped with alkali metals have selective optical absorption in the near-infrared region. This has induced the synthesis of new compounds and various morphologies, including nanorods, nanowires, and nanosheets (Lee et al., 2014). Jeon et al. (2019) studied on ethylene-propylene-diene monomer (EPDM) nanocompounds by synthesizing tungsten bronze nanorods and nanoparticles coated with alkyl chains, and confirmed that mechanical and photothermal properties were enhanced. As such, studies using nanoparticles have mainly been conducted by blending in polymer materials, but that process concern the yarn or fabric stage and not the textile manufacturing stage. So it is necessary to study a post-finishing method in the case of fashion-sensitive materials that require a multi-step process. Therefore, a post treatment process for attaching inorganic nanoparticles to fiber surface was developed. And a silane coupling agent which improves the efficiency of the finishing process and produces a practical and excellent laundry resistance product was introduced to improve the washing durability of outdoor sportswear.

Silane coupling agents contain inorganic reactive sites and bond with most inorganic substrates, including glass, metal, and silica, especially if the substrate contains elements such as silicon, aluminum, and most heavy metals in its structure. If the coupling agent is condensed at the interface, a multi-molecular structure of cross-linked siloxane is generated on the surface of the inorganic material. When the silane coupling agent is attached to the surface of the inorganic material, the surface exhibits surface chemistry or surface reactivity characteristics of organic groups attached to the silane coupling agent. The treated surface shows the surface energy of the aforementioned organic groups, which can be a reactive surface determined by the reactivity of the organic functional groups in the silane coupling agent (Kutz, 2011). Therefore, the coupling agent plays the role of an intermediary, connecting organic and inorganic materials, which are naturally difficult to associate (Song et al., 2011).

Jo et al. (2017) conducted a study to treat the fiber surface with a silane coupling agent during the formation of composite materials, and the results showed that the interfacial shear strength, mechanical properties of the composite materials were improved. Boussehel (2019) studied the thermal and mechanical properties of polystyrene composites using 3-(trimethoxysilyl) propyl methacrylate (TMSPMA) as the silane coupling agent, and it was confirmed that the composite treated with the coupling agent was more thermally stable. Therefore, the silane coupling agent enabled the bonding of difficult-to-bond materials. In this study, TMSPMA is used to treat the tungsten bronze nanorods (TBNRs) on the surface of the polyester fabric, and the physical properties of the resulting material are evaluated.

As the demand for materials in response to environmental changes, various materials with thermal insulation and heating functions are being developed in various forms according to their application (Lee & Song, 1994). There are many studies on photothermal materials; however, there is lack of empirical studies that analyze the change in the physical properties of fibers after attaching photothermal materials to the fiber surface. To apply this technology to various materials and processes at the fabric stage, it is necessary to study the post-finishing method of attaching functional materials to the exterior of the fabrics. In this study, nanorods of tungsten bronze, a photothermal material, were attached on the polyester surface at various concentrations. In addition, TMSPMA was used during the processing of the polyester surface to improve the washing durability of the TBNRs.

## Methods

### Materials

Ammonium metatungstate hydrate (AMT), oleylamine, and 3-(trimethoxysilyl)propyl methacrylate(TMSPMA) were purchased from Sigma–Aldrich. Sodium hydroxide (NaOH) was purchased from Samchun Chemical. Toluene and acetone were purchased from DaeJung chemicals. White 100% polyester fabric (microfiber) was purchased from Dou fabric.

### Preparation of tungsten bronze nanorods (TBNRs)

AMT (2.956 g), oleylamine (160 mL), and NaOH (0.1584 g) were added to a three-neck round-bottomed flask. After connecting a reflux condenser, thermometer, and long needle to the flask, it was stirred by injecting nitrogen gas for 1 h to create a nitrogen atmosphere inside the flask. After 1 h, the flask was heated to 140 °C, followed by heating to 250 °C by increasing the temperature gradually every 10 min. The flask was heated with stirring at 250 °C for 8 h, keeping the temperature below 250 °C. After 8 h, the reaction mixture was cooled to room temperature. The precipitate was collected by performing centrifugation (three times for 15 min at 8000 rpm). It was treated with acetone to remove the excess oleylamine and then dried at room temperature. When alkali metal ions (M = Li^+^, Na^+^, K^+^, Cs^+^) are used to reduce tungsten trioxide, the reduced WO_3_ and M_x_WO_3_ materials strongly absorb NIR and release heat (Park, 2020). So TBNRs were synthesized by the thermal decomposition of AMT in OA.

### Functional finish with TBNRs and silane

All microfiber PET samples were prepared in sizes of 2 × 2 cm, weight of 0.0271 g, and thickness of 0.12 mm, with bath ratio set at 30:1. The fabric coatings of 1, 3, 5, and 10 wt%. TBNRs were prepared by dissolving 0.00813, 0.02439, 0.04065, and 0.0813 g of TBNRs powder in toluene, respectively, and stirred. The PET pieces of the desired size (2 × 2 cm) were immersed in the solution for approximately 1 h. For coating of TBNRs and silane on fabric, 0.04065 g of TBNR powder and 0.00406 g of silane were dissolved in 0.7684 g of toluene, and then the PET pieces of desired size (2 × 2 cm) were stirred in the solution at 400 rpm for 1 h. The concentrations of TBNRs and silane coupling agent were set at 5 and 0.5 wt%, respectively, which were found to produce optimal photothermal effect in the preliminary experiment. The coated fabrics were dried at room temperature for a day, washed with distilled water at 40 °C for 5 min, and conditioned at 21 °C. The pick-up rate of PET with TBNRs is 3.32% and the pick-up rate of PET with TBNRs and silane is 4.06%.

### Characterization

**Fig. 1:**
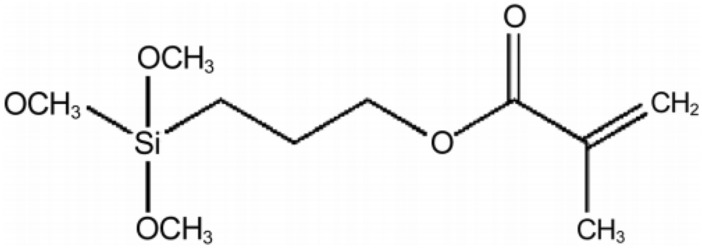
Chemical structure of 3-(trimethoxysilyl)propyl methacrylate(TMSPMA)

The sizes of the TBNRs were analyzed using the Gatan Microscopy Suite (Gatan Inc., Pleasanton, CA, USA) for images measured through FE-TEM (FEI Tecnai G2 F30 S-Twin). Ultraviolet–visible (UV–Vis) absorption spectra were obtained using a UV–Vis spectrometer (V-670, JASCO, Tokyo, Japan) in the range of 300–2100 nm. To determine the influence of TBNRs on the photothermal effect of polyester, the surface temperature change upon irradiation with a solar simulator with white light (100 W, PEC-L01, Peccell Technologies Inc., Yokohama, Japan) was measured. The values of L*, a*, b*, and ΔE were measured thrice in a colorimeter (AMT507) for each of the samples, and the elemental analysis of the samples was carried out by performing field emission scanning electron microscopy with energy dispersive X-ray spectroscopy (FE-SEM–EDX, JSM6701, JEOL, Japan) on the samples (Fig. 1). To compare the tensile properties of the samples before and after finishing, the tensile strength was measured using a tensile strength tester in the warp direction of the fabric according to the ravel strip method (KS K 0520). The test was carried out by preparing a rectangular polyester sample of size 2.5 × 15 cm^2^.

## Results and Discussion

### Synthesis of TBNRs and fabrication of polyester fabric

**Fig. 2:**
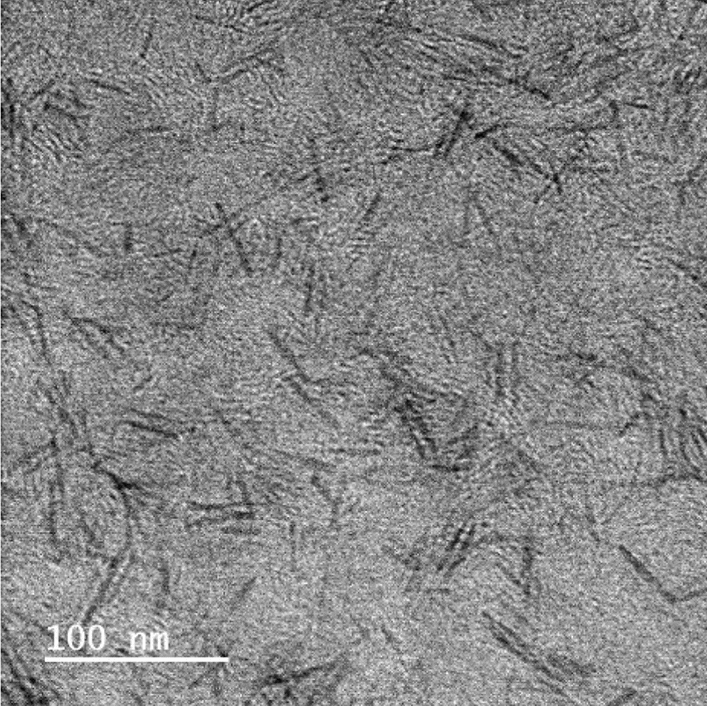
FE-TEM image of tungsten bronze nanorods (TBNRs)

TBNRs were synthesized by the thermal decomposition of AMT in OA, and the formation of nano-sized materials of the synthesized TBNRs was confirmed by image analysis with the FE-TEM. The as-prepared TBNRs samples shown in Fig. 2 indicate that the length is approximately 34.0 ± 2.5 nm and the diameter is approximately 2.4 ± 0.4 nm, and it shows the shape of nanomaterials; hence, they can be considered nanorods.

**Fig. 3:**
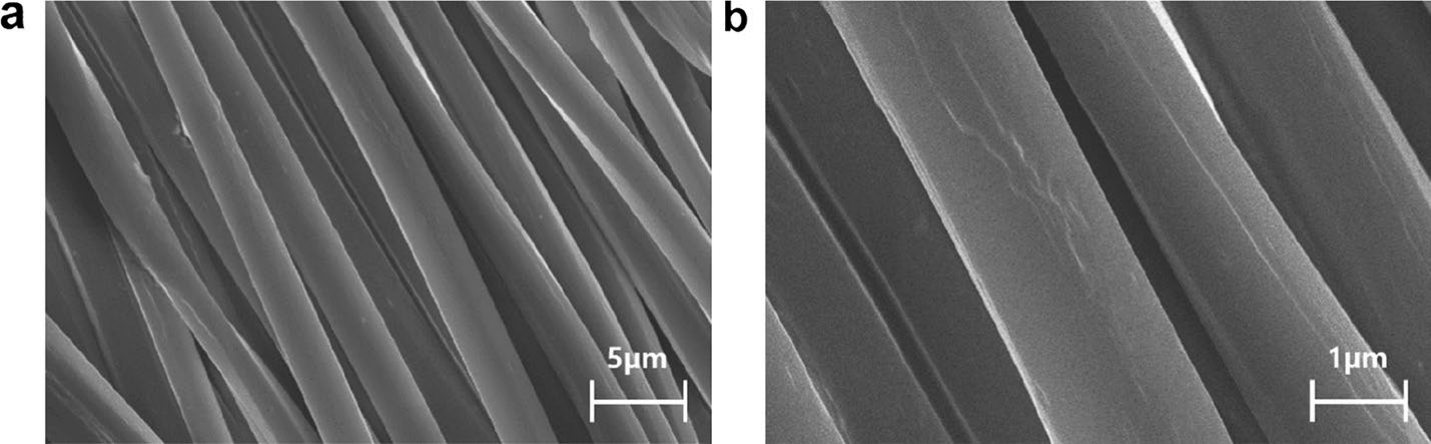
FE-SEM Image of PET fabric treated with tungsten bronze nanorods (TBNRs) 5 wt%. (Magnification **a** × 3.2 k, **b** × 10 k)

**Fig. 4:**
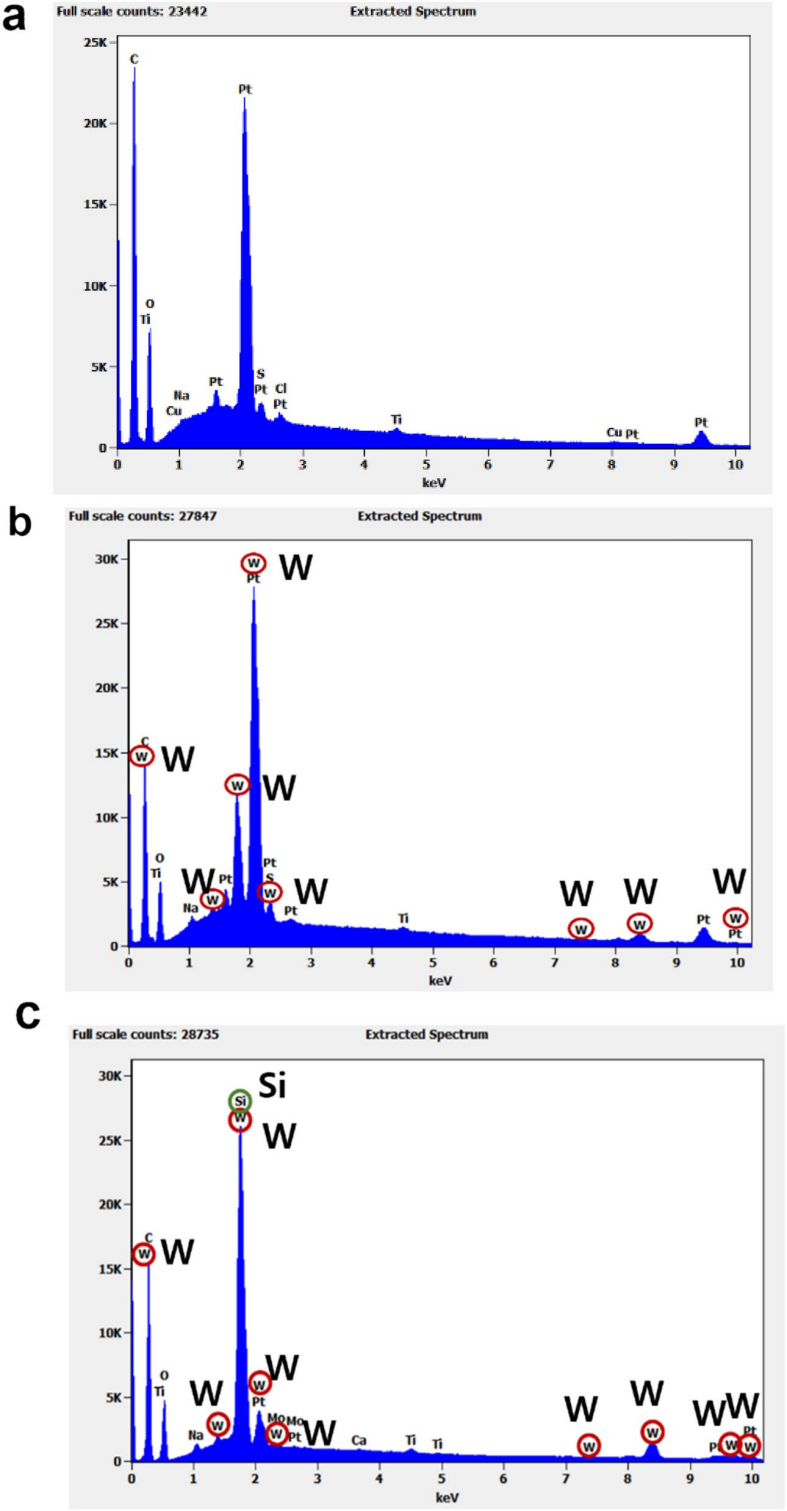
Energy dispersive X-ray spectroscopy (EDS) analysis of PET fabric **a** before treatment with tungsten bronze nanorods (TBNRs), **b** after treatment with TBNRs, and **c** after treatment with TBNRs and silane

FE-SEM and EDS analyses were performed to confirm that the TBNRs were well attached to the polyester fabric. The nanorods on the surface of the fabric could not be identified in the FE-SEM image shown in Fig. 3, since the TBNRs are nano-sized and too small to be measured. Thus, tungsten adhesion was confirmed through EDS analysis, as shown in Fig. 4. Observing the EDS spectra before and after the treatment of polyester fabric, that of the untreated polyester fabric (Fig. 4a) which did not show any characteristic tungsten signal (W) showed only matrix of C, O, Na, S, Cl, Ti, Cu and Pt; however, a clear tungsten signal was observed in spectrum of the polyester treated with TBNRs (Fig. 4b) and a clear tungsten signal and silicon signal were observed in spectra of the polyester with both TBNRs and silane coupling agent (Fig. 4c). And the analysis shows substantial amount of tungsten. These results from EDS analysis through comparison with untreated fabrics clearly indicate that TBNRs are attached on polyester.

### Photothermal effect of polyester fabric treated with TBNRs

The surface temperature of the fabric was measured to investigate whether the TBNRs exhibits a photothermal effect even when treated with polyester. To examine the effect of concentration on the photothermal effect of the polyester fabrics, fabric surface temperature was measured with the solar simulator using NIR camera at 10-min intervals for 1 h. The initial surface temperature of all fabrics was 21 °C.

**Fig. 5:**
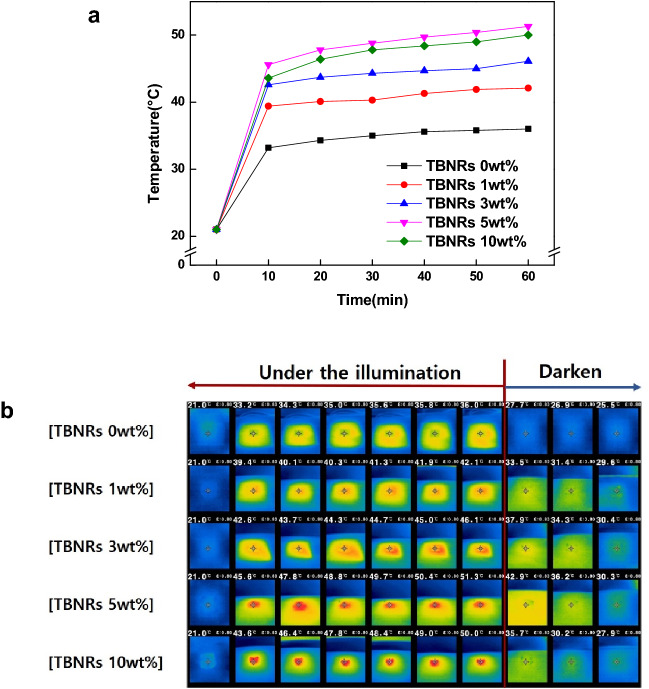
Graph of temperature change of PET samples with time (**a**) and NIR images (**b**) under white light irradiation for 1 h and after turning off the light for the following 15 min

Figure 5 presents the graph and NIR images showing the temperature rise of the polyester fibers samples over time. For each TBNRs concentration (0, 1, 3, 5, and 10 wt%), the rate of temperature increase in the first 10 min after irradiation and the maximum temperature of the polyester after irradiation for 1 h were as follows: 1.2 °C/min, 36.0 °C; 1.8 °C/min, 42.1 °C; 2.2 °C/min, 46.1 °C; 2.5 °C/min, 51.3 °C; and 2.3 °C/min, 50.0 °C. After blocking the light, the fabric surface temperature dropped rapidly and returned to its original temperature within 30 min. The results show that the higher the concentration, the higher the rate of temperature increase and the maximum temperature reached by the fiber. However, Fig. 5 shows that the maximum rising temperature of 5 wt% TBNRs is higher than that of 10 wt% TBNRs. This results show that the most effective concentration of TBNRs was 5 wt%, because the surface temperature of the fabric attained a maximum at this concentration, after which it started to fall. Furthermore, the maximum surface temperature of the fabric with TBNRs treatment was 15.3 °C higher than that of the non-TBNRs-treated fabric.

Tungsten bronze absorbs the broad NIR due to the fact that the ternary addition of cations causes free electrons to absorb dipoles and interact with photons. And tungsten bronze exhibits an exothermic effect since PP (plasmonic polariton) of free electrons has a strong heat absorption ability in the NIR wavelength range (Sang et al., 2020). Therefore, it can be seen that TBNRs demonstrates a photothermal effect even when coated on the fabric surface.

### Effect of the silane coupling agent

**Fig. 6:**
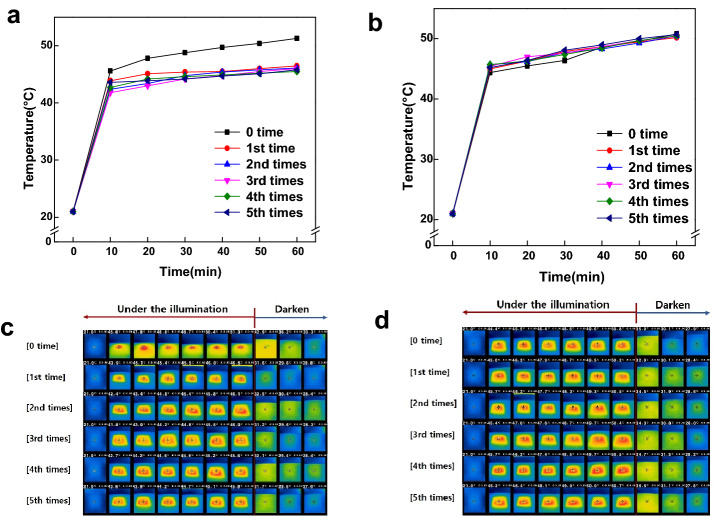
Graph of temperature change of **a** PET with tungsten bronze nanorods (TBNRs) and **b** PET with TBNRs and silane treatment, and NIR images after repeated washing of **c** PET with TBNRs and **d** PET with TBNRs and silane treatment

The photothermal effect of the TBNR-treated fabrics and the fabrics treated with TBNRs and silane coupling agents after multiple washing is shown in Fig. 6. The washing durability was evaluated based on the presence or absence of the silane coupling agent. Figure 6a and b show the changes in temperature measured at 10-min intervals using a solar simulator for the polyester treated with a 5 wt% TBNRs solution, with and without the addition of a silane coupling agent. Figure 6c and d show NIR images of the surface temperature change.

The samples were irradiated with a solar simulator for 60 min, and the change in the surface temperature of the fabric was measured. Figure 6a and c show the temperature rise of the polyester treated with TBNRs only, measured every 10 min for 1 h. After each wash cycle, the polyester temperature rose rapidly during the first 10 min, and then increased slowly for the remaining time. After treatment with TBNRs, the increasing rate of temperature and the maximum temperature of the polyester were 2.5 °C/min, 51.3 °C; 2.3 °C/min, 46.5 °C; 2.1 °C/min, 46.1 °C; 2.1 °C/min, 46.0 °C; 2.2 °C/min, 45.5 °C; and 2.3 °C/min, 45.8 °C. After turning off the light, the temperature rapidly decreased and returned to the initial temperature within 30 min.

Figure 6b and d show the surface temperature rise of the polyester mixed with 5 wt% of TBNRs and 0.5 wt% of the silane coupling agent. As shown in Fig. 6a, the temperature of the polyester increased rapidly during the first 10 min and then slowly increased for the remaining time in all washing cycles. After treatment with TBNRs and silane coupling agent, the rate of temperature increase and the maximum temperature of the polyester were 2.3 °C/min, 50.8 °C; 2.4 °C/min, 50.2 °C; 2.5 °C/min, 50.5 °C; 2.4 °C/min, 50.4 °C; 2.5 °C/min, 50.5 °C; and 2.4 °C/min, 50.7 °C. After turning off the light, the temperature rapidly decreased and returned to the initial temperature within 30 min. The maximum temperature rise before and after washing did not change significantly; however, the light-heating effect is more effectively maintained after repeated washing when a silane coupling agent is added than when treated with TBNRs only.

Silane coupling agent play the role in connecting organic and inorganic materials (Song et al., 2011). By using TMSPMA as the role of silane and treating it on fabric, TBNRs were better attached to the fabric surface, resulting in significant improvement in washing durability. These results indicate that TMSPMA is suitable for the role of Silane, which attached TBNRs on the polyester surface.

In the case of fashion materials, it is important to maintain the color when coated with TBNRs and silane coupling agent on white fabric because the process of dyeing must be carried out to be sold on market. Therefore, the change in the color of the fabric after treatment with TBNRs only and TBNRs and silane coupling agent was evaluated. The colors of the untreated polyester, polyester treated with 5 wt% TBNRs, and the polyester treated with TBNRs and silane coupling agent were measured with a colorimeter. Table 1 shows the values measured. When 5 wt% TBNRs was added, L*, a*, and b* values decreased slightly, but the color change was insignificant. Therefore, even though the TBNRs powder is blue, the addition of the silane coupling agent did not negatively affect the color change, and the color change is not significant even if 5 wt% of TBNRs which have the best photothermal effect are treated with white polyester fabric.

**Table 1:** Color values of PET Control, PET treated with 5 wt% tungsten bronze nanorods (TBNRs), and PET treated with 5 wt% TBNRs and 0.5 wt% silane

Color Parameter	PET control	PET with TBNRs	PET with TBNRs and silane
L*	93.7	90.1	90.5
a*	− 0.9	− 2.2	− 1.4
b*	0.3	− 1.2	− 1.1
ΔE	–	4.1	3.5

**Fig. 7:**
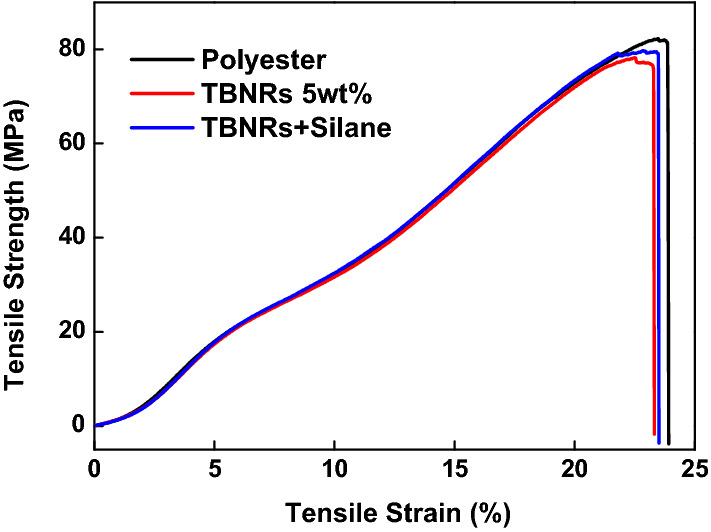
Tensile stress–strain curve of untreated PET, PET treated with tungsten bronze nanorods (TBNRs), and PET treated with TBNRs and silane

In addition, polyester fabric is a fabric that is frequently used as an outdoor fabric, but the use of a large amount of silane coupling agent during the experiment adversely affects properties of polyester. Therefore, in order to determine whether the concentration of the silane coupling agent is appropriate or not, the tensile properties of the treated fabric were measured using the ravel strip method (KS K 0520) to examine the change in physical properties after the fabric processing. The stress–strain curve is presented in Fig. 7.

Figure 7 shows the strength-strain curves of the untreated polyester, polyester treated with 5 wt% TBNRs, and polyester treated with 5 wt% TBNRs with 0.5 wt% silane coupling agent, before and after post-finishing. The figure shows that there is almost no difference in the tensile strength, with or without TBNRs and silane coupling agent, and the sample treated with the silane coupling agent is rather closer to the result of the untreated polyester sample.

Table 2 shows the average values and standard deviations of the breaking strength and breaking elongation of the untreated polyester, polyester treated with 5 wt% TBNRs, and polyester treated with 5 wt% TBNRs and 0.5 wt% silane coupling agent. Similar to the strength-strain curve, there is little difference between the breaking strength and elongation of different samples. Moreover, there was no change in the tensile properties of the sample treated with the silane coupling agent.

**Table 2:** Mechanical properties of PET (control, tungsten bronze nanorods (TBNRs), TBNRs and silane)

Samples	Strength (MPa)		Strain (%)	
	Average	SD	Average	SD
PET control	79.57	3.65	23.08	1.36
PET with TBNRs	79.70	1.86	23.45	0.37
PET with TBNRs and Silane	79.45	0.52	23.81	0.21

This result indicates that the photothermal effect and high washing durability are simultaneously achieved without a significant change in the strength, elongation, and color of the fabric by the post finishing process of TBNRs and silane coupling agent treatment.

## Conclusions

In this study, nano-sized photothermal TBNRs were synthesized by the post-finishing treatment of polyesters with TBNRs and a silane coupling agent. This increased the heat retention capacity of the polyester and produced a practical polyester fabric for outdoor sportswear with a high washing resistance. The TBNRs were successfully prepared as nanoparticles with an average length of 34.0 ± 2.5 nm and a diameter of 2.4 ± 0.4 nm using OA, and they were uniformly adsorbed after fabric treatment, according to FE-SEM and EDS results.

To confirm the photothermal effect of TBNRs, various concentrations of TBNRs were applied on polyester fabrics, and the photothermal effect was measured with a solar simulator. The results showed that as the TBNRs concentration increased, the photothermic effect increased. After 5 wt%, the maximum temperature was reached; therefore, 5% was chosen as the optimal concentration. To examine the effect of the silane coupling agent, it was mixed with a 5 wt% TBNRs and used to treat polyester fabric, followed by repeated washing. The results showed that the washing durability improved when a silane coupling agent was added. In addition, the effect of the treatment of TBNR and silane coupling agent on the tensile properties and color change of the fabric was evaluated. The results showed that the post-processing treatment effectively increased the photothermal effect without a significant change in the physical properties and color of the polyester.

The results of this study can be used as basic data for developing functional fashion materials with active photothermal and insulation properties. In particular, they confirmed the possibility of application as practical thermal insulation materials in a cryogenic environment. It is expected that, along with basic research on nanoparticles with various performances, new fashion materials will be developed to provide convenient products to consumers.

## Data Availability

The data sets used and analyzed during the current study are available from the corresponding author on reasonable request.
